# Peptidomic Characterization and Functional Properties of Goat and Cow Milk Kefir before and after *In Vitro* Digestion

**DOI:** 10.1021/acs.jafc.6c08235

**Published:** 2026-08-11

**Authors:** Rayssa Cruz Lima, Bum Jin Kim, Carini Aparecida Lelis, Brianne Wai, Rohit Kumar, Jelmir Craveiro de Andrade, Marie Biondi Ryan, David C. Dallas, Carlos Adam Conte-Junior

**Affiliations:** † Graduate Program in Veterinary Hygiene (PPGHV), Faculty of Veterinary Medicine, Fluminense Federal University (UFF), Vital Brazil Filho, Niterói, Rio de Janeiro 24230-340, Brazil; ‡ Center for Food Analysis (NAL), Department of Biochemistry (DBq), Chemistry Institute, 28125 Federal University of Rio de Janeiro (UFRJ), Cidade Universitária, Rio de Janeiro, Rio de Janeiro 21941-598, Brazil; § Nutrition Program, School of Nutrition and Public Health, College of Health, 2694 Oregon State University, Corvallis, Oregon 97331, United States

**Keywords:** dairy, microbial processing, mass spectrometry, angiotensin-converting enzyme inhibitory, free-radical scavenging, bovine milk, caprine milk

## Abstract

Kefir is a fermented beverage produced from goat or cow milk. This study evaluated the bioactive potential and peptidomic profile of kefirs from both species before and after *in vitro* digestion. Peptides from undigested, gastric, and intestinal phases were characterized by LC-MS/MS. *In silico* tools identified sequences homologous to known bioactive peptides, with functions such as antihypertensive, immunomodulatory, and anticancer. β-casein was identified as the main precursor protein, with the highest number of peptides identified during the gastric phase. Undigested kefir showed antioxidant activity in ABTS and FRAP assays, and peptide extracts from both kefirs exhibited activity in all antioxidant assays across digestion phases. Antimicrobial activity against *E. coli* and *S. aureus* was observed before digestion for both kefir peptides; however, after digestion, only goat kefir peptides remained active against *E. coli*. This data indicates that kefir peptides are promising bioactive compounds for food and pharmaceutical applications.

## Graphical abstract

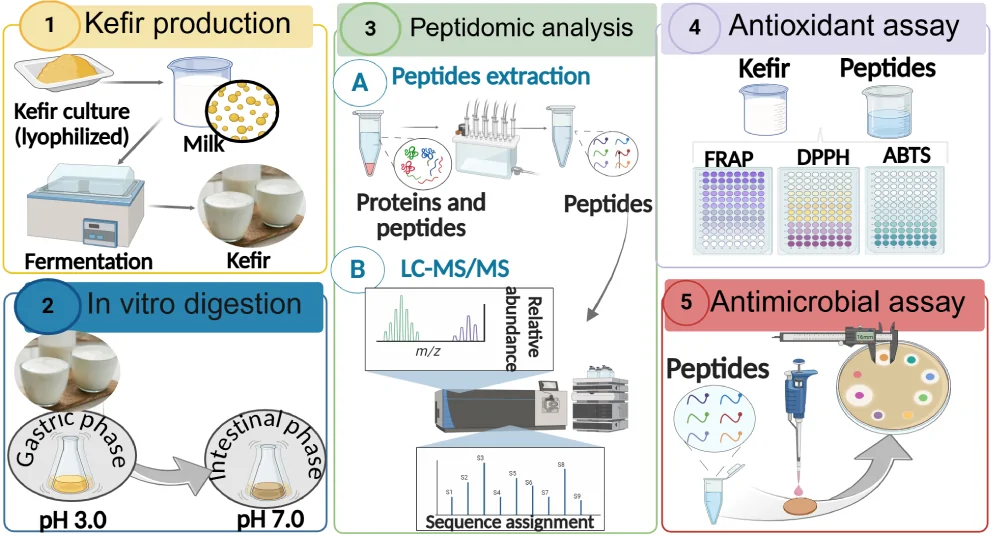

## 1 Introduction

Animal milks provide a wide range of nutrients for human health and can be made into various dairy products.^1^ Kefir, a fermented milk beverage, is produced by fermenting milk, commonly cow or goat milk, with kefir grains, composed of lactic acid bacteria (LAB), yeasts, proteins, and exopolysaccharides.^2^ Industrially, standardized production often employs lyophilized starter cultures.^3^ Fermentation of milk to make kefir involves the metabolic activity of the kefir grain microorganisms, which convert lactose into organic acids and ethanol^4^ and promote proteolysis, leading to the release of peptides from milk proteins.^5^ These peptides, typically 2–20 amino acids in length and with molecular weights <6000 Da,^6,7^ include many with known functions, including antihypertensive,^8–11^ enhanced iron uptake in Caco-2 cells,^12^ antioxidant capacity,^13^ and anticancer.^14^

Cow and goat milks have a similar overall macronutrient composition^15^ but exhibit some differences in protein content that could influence peptide release during kefir production or within the gut after kefir is consumed, which could affect the resulting bioactive peptide functional impacts. Cow’s milk has higher levels of α_s1_-casein (CN; ∼27%) than goat’s milk (∼13%), while goat’s milk has greater proportions of α_s2_-CN (∼16%) than cow’s milk (∼8%).^16,17^ Several studies have investigated the peptide profile of cow milk kefir and demonstrated that the microorganisms present in the culture promote extensive hydrolysis of the milk proteins, complemented by the action of endogenous milk proteases,^4,18,19^ leading to an increase in peptide abundance after fermentation.^20^ In goat milk kefir, fermentation time (0–48 h) has been associated with a progressive increase in antioxidant activity, which is hypothesized to be due to bioactive peptides.^21^ Other investigations evaluated peptides from kefir produced with cow’s milk and reported specific functional properties, such as antihypertensive effects^8,19^ and reductions in Alzheimer’s disease-like symptoms when fed to a *Drosophila melanogaster* model compared with individuals fed an enriched potato powder medium solubilized in distilled water.^22^ Studies found that fermentation-derived compounds in bovine milk kefir exhibit ACE-inhibitory activity, with approximately 63% of this activity retained after *in vitro* gastrointestinal digestion.^23^ Another study reported that ACE-inhibitory activity following *in vitro* digestion was similar to or slightly lower than that observed in nondigested kefir peptides, except for a β-casein peptide (f(47–52)), which exhibited enhanced ACE-inhibitory activity following *in vitro* digestion of goat milk kefir, yielding an abundant peptide hydrolysate (e.g., DKIHP) with an IC_50_ value 8-fold lower than its nonhydrolyzed counterpart (e.g., DKIHPF).^11^

Many studies have evaluated both cow and goat milk kefir; however, it remains unclear how digestion further modifies kefir’s peptide composition and how this differs between the two milk matrices. Digestive conditions (e.g., enzymatic hydrolysis and pH fluctuations) could influence peptide release and functionality. Therefore, identifying the peptides generated during the digestion of cow and goat milk-derived kefir, as well as their biological functions, can provide insights into their potential health effects, such as the bioavailability and biological activity of the released peptides.

Therefore, the aim of this study was to comprehensively characterize the peptide profiles and potential corresponding functions of goat and cow milk kefir before and after *in vitro* gastrointestinal digestion. The overall goal was to provide insights into the potential health-promoting effects produced during the fermentation and digestion of goat and cow milk kefirs.

## 2 Material and Methods

### 2.1 Chemical Reagents

Potassium persulfate, 2,2′-azinobis­(3-ethylbenzothiazoline-6-sulfonic acid) (ABTS), 2,2-diphenyl-1-picrylhydrazyl (DPPH), 6-hydroxy-2,5,7,8-tetramethylchroman-2-carboxylic acid (Trolox), and 2,4,6-Tris­(2-pyridyl)-*s*-triazine (TPTZ) were purchased from Sigma-Aldrich (St. Louis, MO, USA). Potassium chloride (KCl), potassium phosphate monobasic (KH_2_PO_4_), sodium chloride (NaCl), and hydrochloric acid (HCl) were purchased from EMD Millipore Corp. (Billerica, MA, USA). Sodium bicarbonate (NaHCO_3_) and sodium hydroxide (NaOH) were purchased from Fisher Chemical (Fair Lawn, NJ, USA). Magnesium chloride hexahydrate (MgCl_2_(H_2_O)_6_) was purchased from Macron Fine Chemicals (Center Valley, PA, USA). Ammonium carbonate ((NH_4_)_2_CO_3_) was purchased from Acros Organics (Geel, Belgium). Calcium chloride dihydrate (CaCl_2_(H_2_O)_2_) (Fisher Chemical (Cat. No. C79-500). For *in vitro* digestion, pepsin from porcine gastric mucosa (CAS No.: 9001-75-6), pancreatin from porcine pancreas (CAS No.: 8049-47-6) and bovine bile salts (CAS No.: 8008-63-7, B3883) were purchased from Sigma-Aldrich.

### 2.2 Kefir Production

Whole ultrapasteurized goat milk (Meyenberg) and cow milk (Simple Truth Organic) were purchased from a local supermarket (Corvallis, Oregon, USA). Freeze-dried Lactic Culture 50 CXU LyoPro KEFIR was obtained from Codex-ing (Miami, Florida, USA). According to the supplier, the starter culture contains *Lactococcus lactis* subsp. *lactis*, *Lactococcus lactis* subsp. *cremoris*, *Streptococcus salivarius* subsp. *thermophilus*, *Lactococcus lactis* subsp. *lactis biovar* *diacetylactis, Leuconostoc* *cremoris,* and *Kluyveromyces marxianus*. A commercial freeze-dried starter culture was selected to ensure a standardized microbial composition and improve batch-to-batch reproducibility during kefir production. Kefir was produced as previously described, with minor modifications.^24^ Three independent batches of kefir (100 mL) were prepared for each milk type. Fifteen milligrams of lyophilized culture were mixed into 100 mL of cow or goat milk, and all samples were incubated at 25 °C for 21 h without mixing. After fermentation, the kefir reached pH values between 4.2 and 4.3, measured via a pH meter (Hanna Instruments), and samples were stored at 4 °C for 1 day to decrease the culture activity, followed by storage at −20 °C prior to subsequent analyses.

### 2.3 *In Vitro* Gastrointestinal Digestion

Static adult *in vitro* digestion of the kefirs was conducted according to the INFOGEST 2.0 method^25^ for the gastric and intestinal phases for each of the individual batches of kefir.

#### 2.3.1 Gastric Phase

Five milliliters of thawed kefir was mixed with 4 mL of simulated gastric fluid (SGF—at 1.25× initial concentration) previously warmed to 37 °C (as described in Supplementary Table 1). The pH was adjusted to 3.0 with 1 M HCl. Then, 0.3 M CaCl_2_(H_2_O)_2_ was added to obtain a final concentration of 0.15 mM CaCl_2_(H_2_O)_2_ in the SGF already combined with kefir, followed by vortex homogenization. Two hundred and fifty microliters of pepsin (32 mg/mL) were added to obtain a final activity of 2,000 U/mL in the mixture. Ultrapure water was added to reach a final volume of 10 mL, achieving a 0.5× concentration of SGF. The final volume of mixture was incubated at 37 °C for 2 h at 300 rpm in a thermomixer (Eppendorf ThermoMixer C). Afterward, the pH was adjusted to 7.0 with 1 M NaOH, and 5 mL of the sample was collected and frozen at −20 °C for future analysis.

#### 2.3.2 Intestinal Phase

Five milliliters of gastric chyme were mixed with 2.125 mL of simulated intestinal fluid (SIF—at 1.25× initial concentration) previously warmed to 37 °C (described in Supplementary Table 1). The pH of the mixture was adjusted to 7.0 with 1 M NaOH. Then, 0.3 M CaCl_2_(H_2_O)_2_ was added to obtain a final concentration of 0.6 mM CaCl_2_(H_2_O)_2_ in SIF already combined with kefir, followed by vortex homogenization. Six hundred twenty-five microliters of bile salts (97 mg/mL in SIF) were added to obtain a final concentration of 10 mM, and 1.250 mL of pancreatin (258.06 mg/mL in SIF) solution was added to the mixture to obtain a final activity of 100 U/mL. Ultrapure water was added to reach a final volume of 10 mL, resulting in a 0.5× concentration of SIF. The final mixture was incubated at 37 °C for 2 h at 300 rpm in the thermomixer. At the end of the intestinal digestion, 50 μL of 0.1 M Pefabloc SC was added to inhibit proteases, and the sample was stored at −20 °C for future analyses.

### 2.4 Peptidomic Assay

#### 2.4.1 Peptide Extraction from Undigested and Digested Kefir Samples via Ethanol Precipitation

Undigested and digested kefir samples frozen at −20 °C were thawed in a 4 °C fridge overnight in contact with ice. The samples were homogenized with a vortex mixer for 30 s, and 1,500 μL of the samples were transferred to new microcentrifuge tubes in duplicate. These samples were centrifuged (Eppendorf centrifuge 5810R) at 12,000 × *g* for 30 min at 4 °C to separate the supernatant (containing peptides) from the pellet (containing intact protein). Nine hundred microliters of the supernatant from each duplicate were mixed together once again, resulting in 1,800 μL as the new final volume. This sample was subjected to centrifugation again under the same conditions as before, and the supernatant was collected. To remove any remaining intact proteins, 1,500 μL of the supernatant were mixed with ethanol (previously cooled to −20 °C) in a 1:5 (v/v) ratio and stored at −20 °C for 1 h. Samples were centrifuged at 3,214 × *g* for 1 h at 4 °C, and the supernatant was collected, whereas the precipitated protein was discarded. Supernatants were stored at −20 °C for ∼15 h. The solvent was partially evaporated via vacuum centrifugation (Genevac EZ-2.3 Plus). The microtube containing the peptide solution was sealed with parafilm and stored at −80 °C for 24 h. The samples were subjected to freeze-drying (Labconco FreeZone) for 24 h. Dried samples were reconstituted with 1 mL of ultrapure water and stored at −20 °C until future analyses.

#### 2.4.2 Solid-Phase Extraction (SPE) of Peptide Extracts

The samples were thawed in the refrigerator (4 °C) in contact with ice for 24 h. C18 cartridges (Discovery DSC-18 SPE) were coupled to the VacMaster 20 (Biotage), and a vacuum was initiated. Cartridges were reconditioned by adding 5 mL of ultrapure water, 5 mL of 80% acetonitrile (ACN, HPLC grade) with 0.1% trifluoroacetic acid (TFA), and 5 mL of ultrapure water. To load the samples, 1 mL of ultrapure water was added, followed by 1 mL of the sample. To wash off more polar components (salts, sugars, etc.), 5 mL of ultrapure water was added. To elute peptides, 5 mL of 80% ACN with 0.1% TFA was added. All steps were carried out with a flow rate of 1–2 drops per second. Eluents were completely dried via vacuum centrifugation and frozen at −80 °C.

#### 2.4.3 Peptide Characterization via LC-MS/MS

Prior to LC-MS/MS analysis, the dried peptide samples were reconstituted with 600 μL of a solution containing 3% ACN and 0.1% TFA, followed by a 10-fold dilution (with 3% ACN and 0.1% TFA). Peptide separation and detection were performed on a nanoAcquity UPLC system (Waters, Milford, MA, USA) connected to an Orbitrap Fusion Lumos Tribrid mass spectrometer (Thermo Scientific, Waltham, MA, USA). For chromatographic processing, peptides were first enriched and desalted using a nanoEase M/Z Symmetry C18 trap column (180 μm × 20 mm, 5 μm particle size; Waters). Subsequent separation was achieved on a nanoEase M/Z Peptide BEH C18 analytical column (75 μm × 100 mm, 1.7 μm particle size; Waters). One microliter of each sample was injected, with a flow rate of 3 μL/min, and chromatographic separation was conducted over a 60-min run time.

Mobile phases consisted of solvent A (0.1% formic acid in ultrapure water) and solvent B (0.1% formic acid in ACN). The mobile phase gradient was programmed as follows: 3% B (0–3 min), 3–15% B (3–4.5 min), 15–21% B (4.5–25 min), 21–35% B (25–45 min), 35–95% B (45–50 min), 95% B (50–55 min), 95–3% B (55–55.5 min), and 3% B (55.5–60 min). Peptides were ionized via electrospray at 2,350 V, with the transfer tube maintained at 300 °C. Full-scan mass spectra were acquired in positive ion mode over an *m*/*z* range of 300 to 2,000 (MS1), with a resolution of 60,000. Instrument settings included an automatic gain control (AGC) target of 4.0 × 10^5^, with an Orbitrap detector, a maximum injection time of 50 ms, and an MS cycle time of 3 s. Precursor ions selected for MS/MS analysis met the following criteria: ion intensity of at least 5.0 × 10^4^, charge states ranging from 2 to 8, and a dynamic exclusion time of 60 s. Fragmentation was performed using higher-energy collision-induced dissociation (HCD) at 30% normalized collision energy. All MS/MS spectra were acquired in positive ion mode at a resolution of 30,000, with the mass range set to “Normal” with “Auto” scan range mode, using the Orbitrap detector.

#### 2.4.4 Peptidomic Data Processing

Peptide identification was conducted using Proteome Discoverer (v.3.2; Thermo Scientific) with the SequestHT search against an in-house *Bos taurus* cow milk protein database (n = 490, Dallas lab) and *Capra hircus* (goat) protein database (n = 263, UniProt). Oxidation of methionine, phosphorylation of serine and threonine, and acetylation of N-termini were selected as dynamic modifications. Peptide identifications were accepted at a false discovery rate (FDR) of ≤1% (*q* < 0.01). Peptide abundances corresponding to the relative ion intensities were log10-transformed and multiplied by the dilution factor corresponding to each digestion stage before statistical analysis. Samples collected before digestion were multiplied by 10, corresponding to the dilution factor of the aliquots injected into the LC-MS/MS system. Gastric digests were multiplied by 20, reflecting an additional 2-fold dilution relative to the undigested samples (2 × 10). Intestinal digests were multiplied by 40, corresponding to a 4-fold dilution relative to the undigested samples (4 × 10). To correctly interpret these results, it is important to note that in peptidomic analyses, peptide count and peptide abundance represent related but distinct parameters. Peptide count refers to the number of peptides identified within a sample or assigned to a given protein, thereby reflecting the diversity of peptide sequences generated during digestion. In contrast, peptide abundance corresponds to the relative signal intensity of the detected peptides (e.g., peak area or height), which provides information on their relative quantity within the sample. The sequences of the identified peptides and their corresponding proteins are provided in Supporting Information 2.

#### 2.4.5 Bioactive Peptide Search

Peptides from goat and cow milk kefir, prior to and after digestion, were searched for homology against the Milk Bioactive Peptide Database (MBPDB; via https://mbpdb.nws.oregonstate.edu/peptide_search/)^26,27^ to determine their known biological functions. The search criteria included sequence as the search type, with a similarity threshold of 100%, and were not limited to caprine and bovine species alone.

### 2.5 Total Antioxidant Capacity (TAC)

To determine the antioxidant activity of undigested and digested goat and cow milk kefir and peptide extracts (samples obtained after ethanol precipitation, Section 2.4.1), the samples were thawed in a refrigerator at 4 °C overnight, homogenized by vortexing, and used for ABTS, FRAP, and DPPH assays.

#### 2.5.1 ABTS Decolorization Assay

The radical cation scavenging assay (ABTS) was performed as previously described.^28^ The analysis was conducted by adding 190 μL of ABTS working solution into 96-well plates, followed by 10 μL of kefir or extracted peptides (both intact and across digestion) or ethanol (blank). Plates were incubated for 10 min in the dark without shaking and then read at 734 nm by a microplate reader (SpectraMax M2). The average absorbance of the samples was used to calculate the percentage of decolorization according to the following eq 1:

1 $$ABTS\;radical\;scavenging\;activity\;(\%)=\left(\frac{Abs_{control}-Abs_{samples}}{Abs_{control}}\right)\times 100$$

where *Abs_control_* is the absorbance of the ABTS working solution, and *Abs_samples_* is the absorbance of the ABTS working solution with added kefir and or peptides extracted from cow and goat kefir.

#### 2.5.2 Ferric-Reducing Antioxidant Power (FRAP) Assay

The FRAP assay was carried out as previously described.^29,30^ Nine microliters of kefir or extracted peptide (both intact and across digestion) were mixed with 27 μL of ultrapure water, followed by the addition of 270 μL of FRAP reagent (0.3 M acetate buffer, pH 3.6; 10 mM TPTZ solution; and 20 mM ferric chloride, homogenized in a ratio of 10:1:1 (v/v/v)). The samples were kept at 37 °C using a thermomixer for 30 min in the absence of light and without shaking. Absorbance was measured at 595 nm by a microplate reader (SpectraMax M2). A standard curve was constructed using Trolox as the standard reagent. A Trolox stock solution was prepared at a concentration of 1,600 μM, and the standard curve ranged from 160 to 1,600 μM/L. FRAP antioxidant activity was expressed as μM of Trolox equivalents per 100 mL of sample (μM TE 100 mL^–1^).

#### 2.5.3 DPPH Free Radical Scavenging Activity

The antioxidant capability of kefir and peptides extracted from kefir in scavenging DPPH free radicals was determined according to previously described methods.^28^ DPPH (0.1 mM) was prepared in 50 mL of ethanol. One hundred twenty microliters of DPPH solution were added into the wells of a 96-well plate and mixed with 80 μL of the samples. The plate was incubated in the dark for 30 min at room temperature. Absorbance was measured at 515 nm by a microplate reader (SpectraMax M2). Ethanol was used as the control. The percentage of DPPH radical scavenging activity was calculated using the following eq 2:

2 $$DPPH\;radical\;scavenging\;activity\;(\%)=\frac{\left(Abs_{control}-Abs_{sample}\right)}{Abs_{control}}\times 100$$

where *Abs_control_* is the absorbance of the ethanol blank, and *Abs_sample_* is the absorbance of the DPPH solution with added kefir and or extracted peptides from kefir (both intact and across digestion).

### 2.6 Antimicrobial Activity

Antimicrobial activity of peptides extracted from undigested and digested kefir was assayed by the disk diffusion method, using *Escherichia coli* ATCC 25922 and *Staphylococcus aureus* ATCC 29213 as representative Gram-negative and Gram-positive bacteria, respectively. Previously, colonies were picked from culture plates not older than 48 h, and a 0.5 McFarland turbidity equivalent suspension of test bacteria was prepared using the colony suspension method. To obtain a uniform lawn of the test pathogen, a cotton swab was dipped into the resulting suspension and evenly spread on the Mueller-Hinton Agar surface. Sterile 6 mm paper disks (BBL) were placed onto the agar surface. Then, 40 μL of samples were transferred onto the paper disk. One microliter of streptomycin (50 mg/mL) was used as the positive control, and PBS (1 μL) was used as the negative control. Plates were kept at 37 °C overnight, and the inhibition zone (the halo with no bacterial growth) diameter (mm) was measured.

### 2.7 Statistical Analysis

Goat and cow milk kefirs were produced in three independent batches (biological triplicates). Data are reported as mean ± standard deviation. Normally distributed data were analyzed by one-way ANOVA and Tukey’s post hoc tests. Data with a non-normal distribution were analyzed with the nonparametric Kruskal–Wallis test with pairwise comparison by Steel-Dwass. Differences were considered significant at *p* < 0.05 and were indicated by different letters. Venn diagrams were created by InteractiVenn (https://www.interactivenn.net/). All analyses were performed with JMP 18 Statistical Software and Minitab 19. For the bar chart, Python version 3.14 was employed, using Pandas for data analysis and Plotly for graph generation.

## 3 Results and Discussion

### 3.1 pH Value

Sample pH decreased from before to after the fermentation process. Before the addition of kefir culture, the pH of goat milk was 6.60, and cow milk was 6.70. After 21 h of fermentation at 25 °C, the pH was 4.23 ± 0.06 for goat kefir and 4.27 ± 0.06 for cow kefir. This observed postfermentation pH is relatively consistent with other studies of kefir, albeit slightly lower.^3,31,32^ In previous studies, goat kefir produced under two temperature conditions—at room temperature and 37 °C with 24 h of fermentation—reached final pH values of 4.34 and 4.33, respectively,^31^ and cow kefir made with starter culture, incubated at 24 °C for 24 h, had a pH of 4.61.^3^ Another study indicated that the pH of cow and goat milk kefir incubated at 25 °C stabilized between 4.5 and 4.6, with no significant differences observed between kefir types.^32^

### 3.2 Undigested and *In Vitro*-Digested Goat and Cow Kefir Peptide Profiles

Goat and cow kefirs produced unique peptide profiles before and during digestion. In goat kefir (Figure 1A), a total of 452, 476, and 23 peptides were identified in the undigested, gastric, and intestinal phases, respectively. Among these, 11 peptides were consistently detected across all three digestion stages, indicating modifications in the peptide profile during gastrointestinal digestion. In contrast, cow kefir (Figure 1B) showed a higher number of peptides identified in both digestion phases but fewer peptides identified in the undigested kefir, with 417, 485, and 107 peptides detected in the undigested, gastric, and intestinal samples, respectively. A higher number of peptides was shared among all three phases (50 peptides) for cow kefir than for goat kefir.

**Figure 1:**
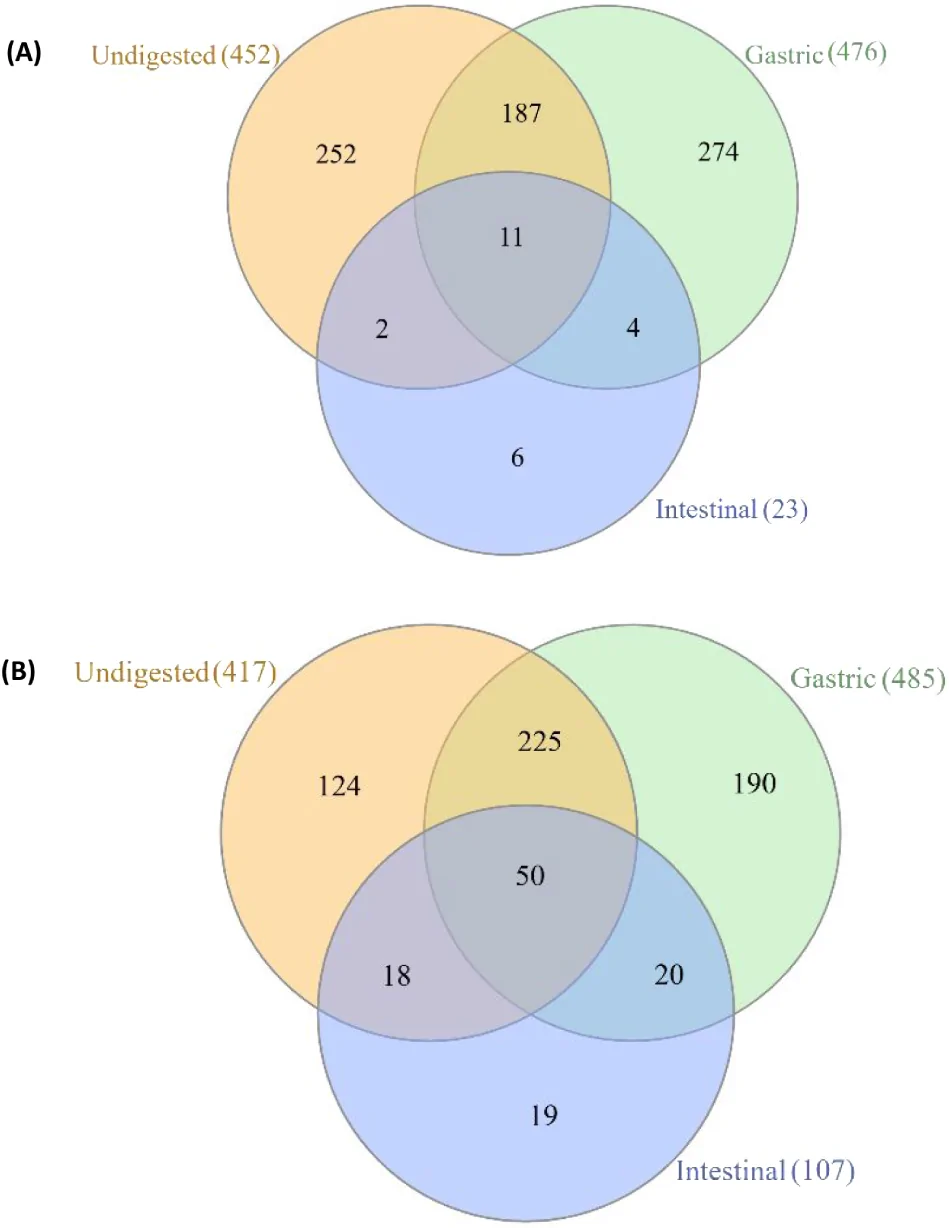
Peptide count at the undigested, gastric, and intestinal phases for goat kefir peptides (A) and cow kefir peptides (B).

#### 3.2.1 Peptide Count

In both goat and cow kefirs, peptides originating from both casein and whey proteins were identified (Figure 2A and 2B**)**.

**Figure 2:**
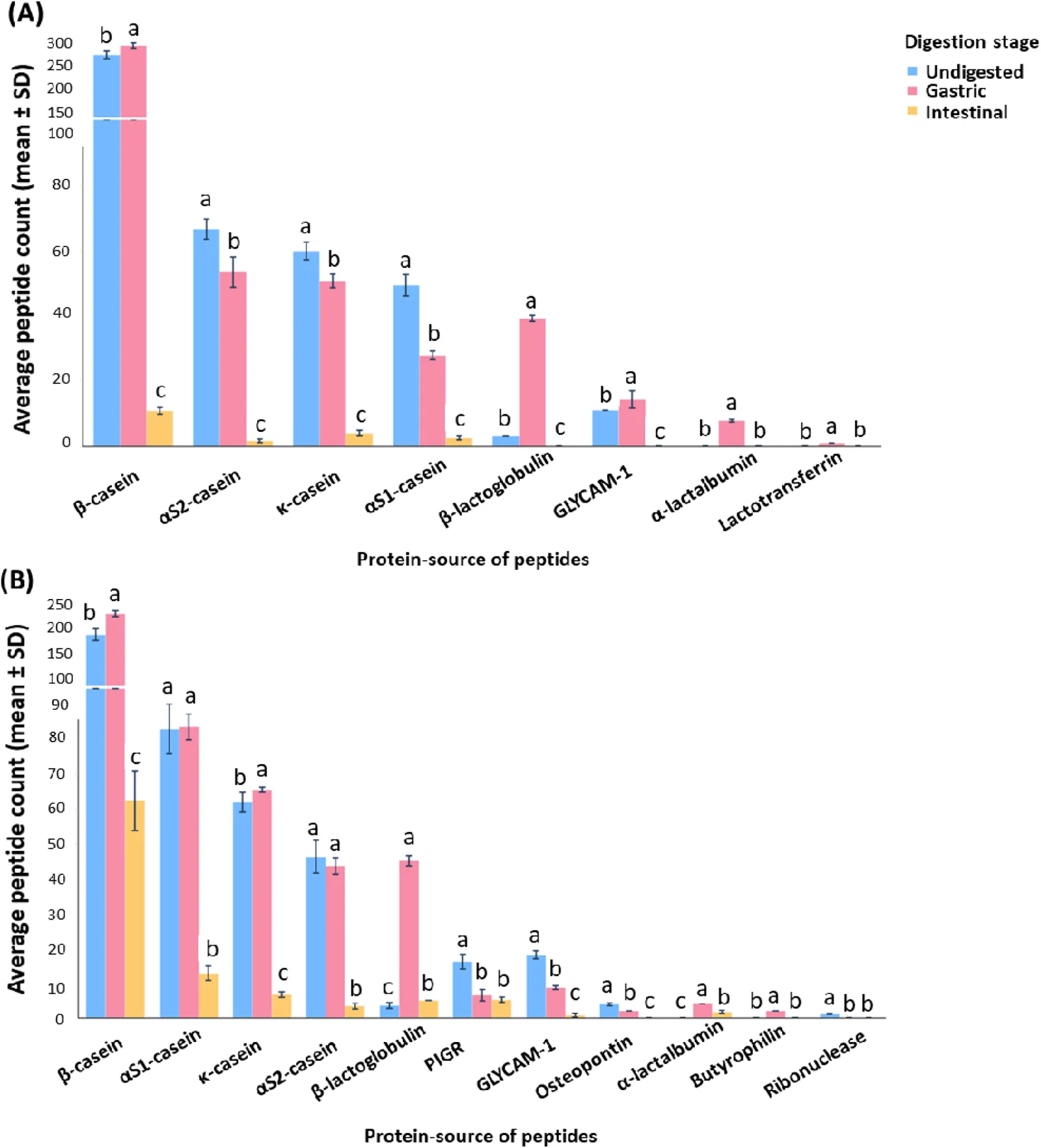
Average peptide count of goat kefir (A) and cow kefir (B) across *in vitro* digestion. GLYCAM-1: Glycosylation-dependent cell adhesion molecule 1; PIGR: Polymeric immunoglobulin receptor. For each protein source, the undigested, gastric, and intestinal phases within a single protein with different letters (a, b, c) were significantly different (*p* < 0.05). Data are reported as mean ± standard deviation.

Before exposure to gastrointestinal digestion, both goat and cow kefir samples exhibited a high number of peptides (Figure 1A and 1B; Figure 2A and 2B). Although raw milk peptides were not analyzed in the present study, a previous study has demonstrated that kefir fermentation markedly enhances peptide generation compared with unfermented cow’s milk.^33^ Initial peptide counts we observed in the kefir result from the proteolytic activity of kefir-associated microorganisms, whose enzymes partially degrade milk proteins during fermentation,^34^ plus some additional contribution from native milk proteases.^27^ In both cow- and goat-derived kefir, β-CN was the major source of peptides. After gastric digestion, β-CN remained the protein source contributing the highest number of unique peptides in both kefir types, with a significant increase from the feed. The number of peptides from β-CN decreased in the intestinal phase. The predominance of peptides derived from caseins should be interpreted in the context of the natural protein composition of milk. Caseins account for approximately 80% of total milk proteins in both bovine and goat milk, whereas whey proteins comprise the remaining protein fraction. Therefore, the higher peptide counts observed for β-CN and the other caseins likely reflect not only their susceptibility to proteolysis during fermentation and simulated gastrointestinal digestion, but also their greater abundance in the original milk. In goat kefir, α_s2_-CN, κ-CN, and α_s1_-CN had the next highest numbers of peptides (in that order), and peptide counts from each decreased from intact kefir to gastric and to the intestinal phase (*p* < 0.05). For cow kefir, the proteins with the next highest counts were α_s1_-CN and α_s2_-CN (in that order), and for each, no decreases in peptide counts were observed from undigested to gastric phase, but decreases were observed in the intestinal phase. In cow kefir, peptide counts for whey proteins (PIGR, GLYCAM-1, osteopontin, and ribonuclease) were significantly higher in the undigested step (*p* < 0.05) than in the gastric and intestinal phases, but this count pattern was not observed for goat kefir, where only peptides originating from GLYCAM-1 were identified, and these were significantly higher in the gastric phase compared with the undigested samples (*p* < 0.05). The lower peptide counts observed for whey proteins are consistent with their lower natural abundance in milk. Peptide counts from α_s2_-CN were higher in goat kefir than in cow kefir in the gastric phase, probably due to the compositional differences of the different milks.^35^ Among whey proteins, β-lactoglobulin was the most abundant contributor of peptides in terms of counts in both cow and goat kefir, with a trend of increasing abundance during gastric digestion (*p* < 0.05).

Our findings align with a previous study examining another fermented milk product, yogurt, in which over 80% of cow and goat yogurt peptides originated from major milk proteins, including α_s1_-CN, α_s2_-CN, and β-CN.^36^ Our findings align somewhat with a previous study that demonstrated kefir produced with cow’s milk (1.5% fat) and fermented using a culture of LAB and yeasts yielded, after *in vitro* gastrointestinal digestion, a peptide profile predominantly composed of β-CN (76%) and κ-CN (24%) fragments. However, unlike in our study, they detected no peptides originating from α_s1_-CN, α_s2_-CN, and β-lactoglobulin.^37^ It is likely that the past study did not detect peptides from the other major milk proteins because the method was less sensitive than the method employed herein (they detected far fewer peptides overall). Our study detected a higher number of peptides in undigested cow milk kefir than a previous study by Ebner et al. (417 vs 230),^38^ although the kefir in their study was produced using kefir grains. Like in our study, Ebner et al. found the undigested cow kefir peptidome was dominated by β-CN-derived peptides, but unlike our study, they detected no peptides originating from whey proteins,^38^ likely due to less sensitive methods used. The predominance of casein-derived peptides compared with whey protein-derived peptides in our study and past studies can be attributed to the higher susceptibility of caseins to enzymatic hydrolysis due to their flexible structure compared with the more compact, globular structure of whey proteins, which makes them more resistant to proteolytic action by microbial enzymes.^38^

#### 3.2.2 Peptide Abundances

Peptide abundances increased for many proteins from the undigested samples (both cow and goat kefirs) to the gastric phase, likely indicating further release of peptides from remaining intact milk proteins, and typically decreased from the gastric to the intestinal phase, likely indicating further proteolysis resulting in amino acids or peptides shorter than those detectable via the applied peptidomic method (<4 AA) (Figure 3A and 3B).

**Figure 3:**
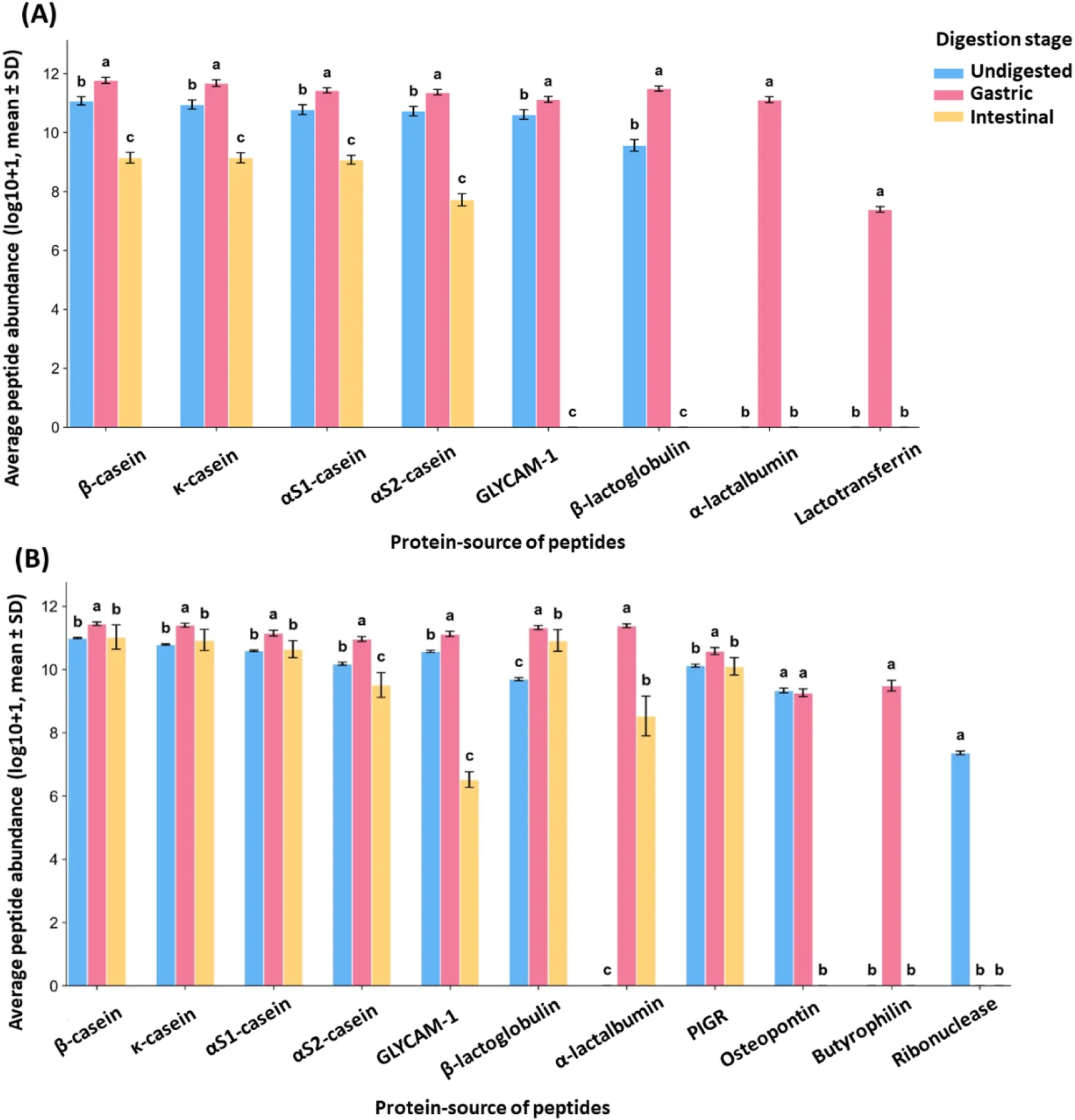
Average peptide abundance (log-10 transformation) of goat (A) and cow (B) kefir across *in vitro* digestion.GLYCAM-1: Glycosylation-dependent cell adhesion molecule 1; PIGR: Polymeric immunoglobulin receptor. Within each protein source, the undigested, gastric, and intestinal phases with different letters (a, b, c) were significantly different (*p* < 0.05). Data are reported as mean ± standard deviation.

Before digestion, the average peptide abundance was significantly lower than during the gastric phase (*p* < 0.05) for almost all protein sources, with the exception of osteopontin and ribonuclease in cow kefir samples. The peptides present in the undigested samples are the result of milk protein cleavage by proteases produced by fermentation microbes or endogenous milk proteases (e.g., plasmin and cathepsin B).^27,39^

The increased abundance of peptides in the gastric sample is likely due to further degradation of milk proteins into peptides by the gastric enzyme pepsin, with some possible additional contributions from the already present endogenous milk proteases and microbial enzymes. After gastric digestion of goat kefir (Figure 3A), peptides from β-CN had the highest abundance. A comparable pattern was observed in cow kefir (Figure 3B). Similarly, Liu and Pischetsrieder observed an increase in β- and κ-CN peptide abundance after the gastric phase from cow kefir, suggesting their high susceptibility to proteolysis.^37^ Very few peptides were detected for the whey protein α-lactalbumin in either the cow or goat kefir, but the abundance of α-lactalbumin-derived peptides was present at high levels in the gastric phase. This finding indicates that α-lactalbumin is resistant to proteolysis by kefir organisms but sensitive to pepsin.

The decrease in the abundance of peptides in the intestinal phase likely results from further digestion of the peptides released in the gastric phase by the added pancreatic proteases (e.g., trypsin, chymotrypsin), resulting in an increase in very short peptides (<4 AA), which could not be identified using the specific peptidomic method employed. In goat kefir, the intestinal peptidome was dominated by peptides from β-, κ-, and α_s1_-CN. Cow kefir had a broader base of relatively abundant protein sources for peptides in the intestinal phase, including β-, κ-, and α_s1_-CN, as well as β-lactoglobulin, α-lactalbumin, α_s2_-CN and PIGR.

### 3.3 Exploring Bioactive Peptides from Goat and Cow Kefir across *In Vitro* Digestion

Undigested goat and cow milk kefir contained an array of peptides with previously identified functions (64 peptides in goat kefir (Table 1) and 152 peptides in cow kefir (Table 2)). The gastrointestinal digestion process increased the number of functional category peptides from cow kefir (65 and 24 for gastric and intestinal, respectively) (Figure 4). For goat kefir, 28 bioactive peptides were identified in the gastric phase, whereas no peptides were identified in the intestinal phase.

**Table 1:** Bioactive Peptides from Goat Kefir across *In Vitro* Digestion

Activity	Protein ID	Precursor protein	Peptide sequence	Detected during digestion	Aligned species (100%)
ACE-inhibitory	P33048	_109_β-casein_120_	GVPKVKETMVPK	Undigested	*Capra hircus*
		_72_β-casein_83_	LVYPFTGPIPN	Undigested and gastric	*Capra hircus*
		_109_β-casein_121_	GVPKVKETMVPKH	Undigested	*Capra hircus*
		_196_ β-casein_203_	RDMPIQAF	Undigested and gastric	*Bos taurus*
		_148_β-casein_154_	LHLPLPL	Undigested and gastric	*Bos taurus*
		_206_β-casein_215_	YQEPVLGPVR	Undigested	*Bos taurus*
		_124_β-casein_133_	MPFPKYPVEP	Undigested	*Bos taurus*
		_208_β-casein_219_	EPVLGPVRGPFP	Undigested	*Bos taurus*
	P18626	_33_α_s1_-casein_38_	ENLLRF	Undigested	*Capra hircus*
		_117_α_s1_-casein_124_	KKYNVPQL	Undigested and gastric	*Ovis aries*
	P33049	_105_α_s2_-casein_111_	YQKFPQY	Undigested and gastric	*Capra hircus*
		_189_α_s2_-casein_194_	KFAWPQ	Undigested and gastric	*Capra hircus*
		_218_α_s2_-casein_223_	PYVRYL	Undigested and gastric	*Bos taurus*
	P02679	_64_κ-casein_70_	YQQRPVA	Gastric	*Ovis aries*
	P00712	_116_α-lactalbumin_122_	DKVGINY	Gastric	*Bos taurus*
	P02756	_28_β-lactoglobulin_37_	LDIQKVAGTW	Gastric	*Bubalus bubalis*
Anticancer	P33048	_95_β-casein_103_	TPVVVPPFL	Undigested and gastric	*Bos mutus*
	P18626	_105_α_s1_-casein_111_	RYLGYLE	Undigested	*Bos taurus*
		_105_α_s1_-casein_110_	RYLGYL	Undigested and gastric	*Bos taurus*
Antimicrobial	P33048	_206_β-casein_220_	YQEPVLGPVRGPFPI	Undigested and gastric	*Capra hircus*
	P33049	_218_α_s2_-casein_223_	PYVRYL	Undigested and gastric	*Bos taurus*
	P02670	_151_κ-casein_160_	PTVHSTPTTE	Undigested and gastric	*Capra hircus*
		_96_β-casein_106_	PVVVPPFLQPE	Gastric	*Capra hircus*
		_76_β-casein_87_	PFTGPIPNSLPQ	Gastric	*Capra hircus*
	P33049	_180_α_s2_-casein_185_	LKKISQ	Gastric	*Ovis aries*
		_127_κ-casein_145_	MAIPPKKDQDKTEVPAINT	Gastric	*Capra hircus*
		_162_κ-casein_174_	IVNTVDNPEASSE	Gastric	*Capra hircus*
	P02756	_61_β-lactoglobulin_73_	VEELKPTPEGNLE	Gastric	*Capra hircus*
Antioxidant	P33048	_93_β-casein_106_	TQTPVVVPPFLQPE	Undigested and gastric	*Bos taurus*
		_126_β-casein_134_	FPKYPVEPF	Undigested and gastric	*Bos taurus*
		_206_β-casein_215_	YQEPVLGPVR	Undigested	*Bos taurus*
	P18626	_105_α_s1_-casein_111_	RYLGYLE	Undigested	*Bos taurus*
	P33049	_105_α_s2_-casein_111_	YQKFPQY	Undigested and gastric	*Capra hircus*
		_96_α_s2_-casein_104_	KALNEINQF	Undigested and gastric	*Bubalus bubalis*
		_218_α_s2_-casein_223_	PYVRYL	Undigested and gastric	*Bos taurus*
	P02670	_117_κ-casein_127_	ARHPHPHLSFM	Undigested	*Bos taurus*
		_50_κ-casein_62_	QYVLSRYPSYGLN	Undigested	*Bos taurus*
Antithrombotic	P33048	_206_β-casein_215_	YQEPVLGPVR	Undigested	*Bos taurus*
DPP-IV Inhibitory	P02670	_72_κ-casein_81_	INNQFLPYPY	Undigested and gastric	*Capra hircus*
Immunomodulatory	P33048	_206_β-casein_215_	YQEPVLGPVR	Undigested	*Bos taurus*
Opioid	P18626	_105_α_s1_-casein_110_	RYLGYL	Undigested and gastric	*Bos taurus*

**Table 2:** Bioactive Peptides from Cow Kefir across *In Vitro* Digestion

Activity	Protein ID	Precursor protein	Peptide sequence	Detected during digestion	Aligned species (100%)
ACE-inhibitory	P02666	_184_β-casein_190_	KVLPVPQ	Undigested and gastric	*Bos taurus*
		_181_β-casein_190_	SQSKVLPVPQ	Undigested and gastric	*Bos taurus*
		_88_β-casein_97_	NIPPLTQTPV	Undigested, gastric and intestinal	*Bos taurus*
		_21_β-casein_29_	LNVPGEIVE	Undigested, gastric and intestinal	*Bos taurus*
		_160_β-casein_169_	HQPHQPLPPT	Undigested and intestinal	*Bos taurus*
		_198_β-casein_205_	RDMPIQAF	Undigested and gastric	*Bos taurus*
		_148_ β-casein_154_	LHLPLPL	Undigested, gastric and intestinal	*Bos taurus*
		_158_β-casein_169_	WMHQPHQPLPPT	Undigested and gastric	*Bos taurus*
		_74_β-casein_83_	VYPFPGPIPN	Undigested, gastric and intestinal	*Bos taurus*
		_209_β-casein_224_	QEPVLGPVRGPFPIIV	Undigested and gastric	*Bos taurus*
		_147_β-casein_155_	NLHLPLPLL	Undigested and gastric	*Bos taurus*
		_160_β-casein_175_	HQPHQPLPPTVMFPPQ	Undigested and gastric	*Bos taurus*
		_210_β-casein_221_	EPVLGPVRGPFP	Undigested and gastric	*Bos taurus*
		_208_β-casein_224_	YQEPVLGPVRGPFPIIV	Undigested and gastric	*Bos taurus*
		_208_β-casein_217_	YQEPVLGPVR	Undigested and gastric	*Bos taurus*
		_73_β-casein_87_	LVYPFPGPIPNSLPQ	Undigested	*Bos taurus*
		_124_β-casein_133_	MPFPKYPVEP	Undigested	*Bos taurus*
		_208_β-casein_218_	YQEPVLGPVRG	Undigested	*Ovis aries*
	P02662	_33_α_s1_-casein_38_	ENLLRF	Undigested, gastric and intestinal	*Capra hircus*
	P02663	_204_α_s2_-casein_212_	AMKPWIQPK	Undigested and gastric	*Bos taurus*
		_104_α_s2_-casein_113_	YQKFPQYLQY	Undigested and gastric	*Bos taurus*
	P02666	_145_β-casein_155_	VENLHLPLPLL	Gastric	*Bos taurus*
	P02662	_16_α_s1_-casein_24_	RPKHPIKHQ	Gastric	*Bos taurus*
	P02663	_104_α_s2_-casein_110_	YQKFPQY	Gastric	*Capra hircus*
	P02754	_28_β-lactoglobulin _35_	IQKVAGTW	Gastric	*Bos taurus*
		_26_β-lactoglobulin_35_	LDIQKVAGTW	Gastric	*Bubalus bubalis*
	P02666	_76_β-casein_83_	PFPGPIPN	Intestinal	*Bos taurus*
		_75_β-casein_81_	YPFPGPI	Intestinal	*Bos taurus*
		_73_β-casein_81_	LVYPFPGPI	Intestinal	*Bubalus bubalis*
Antianxiety	P02666	_75_β-casein_81_	YPFPGPI	Intestinal	*Bos taurus*
Anticancer	P02666	_208_β-casein_224_	YQEPVLGPVRGPFPIIV	Undigested and gastric	*Bos taurus*
		_95_β-casein_103_	TPVVVPPFL	Undigested, gastric and intestinal	*Bos mutus*
	P02666	_75_β-casein_81_	YPFPGPI	Intestinal	*Bos taurus*
Antimicrobial	P02666	_109_β-casein_121_	GVSKVKEAMAPKH	Undigested, gastric and intestinal	*Bos taurus*
		_208_β-casein_224_	YQEPVLGPVRGPFPIIV	Undigested and gastric	*Bos taurus*
		_113_β-casein_120_	VKEAMAPK	Undigested and gastric	*Bos taurus*
		_208_β-casein_222_	YQEPVLGPVRGPFPI	Undigested and gastric	*Capra hircus*
		_56_β-casein_67_	TEDELQDKIHPF	Undigested, gastric and intestinal	*Capra hircus*
	P02662	_114_α_s1_-casein_124_	LRLKKYKVPQL	Undigested and gastric	*Bos taurus*
		_109_α_s1_-casein_115_	YLEQLLR	Undigested	*Bos taurus*
	P02663	_213_α_s2_-casein_222_	TKVIPYVRYL	Undigested and gastric	*Bos taurus*
	P02666	_96_β-casein_106_	PVVVPPFLQPE	Gastric and intestinal	*Capra hircus*
	P02663	_179_α_s2_-casein_184_	LKKISQ	Gastric	*Ovis aries*
Antioxidant	P02666	_157_β-casein_169_	SWMHQPHQPLPPT	Undigested and gastric	*Bos taurus*
		_74_β-casein_83_	VYPFPGPIPN	Undigested, gastric and intestinal	*Bos taurus*
		_181_β-casein_197_	SQSKVLPVPQKAVPYPQ	Undigested and gastric	*Bos taurus*
		_93_β-casein_106_	TQTPVVVPPFLQPE	Undigested, gastric and intestinal	*Bos taurus*
		_208_β-casein_217_	YQEPVLGPVR	Undigested and gastric	*Bos taurus*
		_113_β-casein_120_	VKEAMAPK	Undigested and gastric	*Bos taurus*
		_121_β-casein_138_	HKEMPFPKYPVEPFTESQ	Undigested and gastric	*Bos taurus*
	P02662	_191_α_s1_-casein_207_	APSFSDIPNPIGSENSE	Undigested and gastric	*Bos taurus*
		_194_α_s1_-casein_205_	FSDIPNPIGSEN	Undigested	*Bos taurus*
	P02663	_95_α_s2_-casein_103_	KALNEINQF	Undigested and gastric	*Bubalus bubalis*
		_105_α_s2_-casein_111_	YQKFPQY	Gastric	*Capra hircus*
	P02668	_117_κ-casein_127_	ARHPHPHLSFM	Undigested and gastric	*Bos taurus*
		_72_κ-casein_86_	INNQFLPYPYYAKPA	Undigested and gastric	*Bos taurus*
		_87_κ-casein_98_	AVRSPAQILQWQ	Undigested	*Bos taurus*
		_50_κ-casein_62_	QYVLSRYPSYGLN	Undigested	*Bos taurus*
	P02754	_58_β-lactoglobulin_70_	YVEELKPTPEGDL	Gastric	*Bubalus bubalis*
	P02666	_75_ β-casein_81_	YPFPGPI	Intestinal	*Bos taurus*
Antithrombotic	P02666	_208_ β-casein_224_	YQEPVLGPVRGPFPIIV	Undigested and gastric	*Bos taurus*
		_208_β-casein_217_	YQEPVLGPVR	Undigested and gastric	*Bos taurus*
Cytotoxic	P02754	_17_β-lactoglobulin_24_	LIVTQTMK	Undigested and gastric	*Bos taurus*
DPP-IV Inhibitory	P02666	_84_β-casein_92_	SLPQNIPPL	Undigested, gastric and intestinal	*Bos taurus*
	P02668	_72_κ-casein_81_	INNQFLPYPY	Undigested and gastric	*Capra hircus*
	P02754	_62_β-lactoglobulin_70_	LKPTPEGDL	Gastric	*Bos taurus*
		_28_β-lactoglobulin_35_	IQKVAGTW	Gastric	*Bos taurus*
		_62_β-lactoglobulin_71_	LKPTPEGDLE	Gastric	*Bos taurus*
Immunomodulatory	P02666	_184_β-casein_190_	KVLPVPQ	Undigested and gastric	*Bos taurus*
		_158_β-casein_169_	WMHQPHQPLPPT	Undigested and gastric	*Bos taurus*
		_160_β-casein_175_	HQPHQPLPPTVMFPPQ	Undigested and gastric	*Bos taurus*
		_208_β-casein_224_	YQEPVLGPVRGPFPIIV	Undigested and gastric	*Bos taurus*
		_208_β-casein_217_	YQEPVLGPVR	Undigested and gastric	*Bos taurus*
	P02666	_75_β-casein_81_	YPFPGPI	Intestinal	*Bos taurus*
Increase mucin secretion	P02666	_75_β-casein_81_	YPFPGPI	Intestinal	*Bos taurus*
Opioid	P02666	_75_β-casein_81_	YPFPGPI	Intestinal	*Bos taurus*
Osteoanabolic	P02663	_130_α_s2_-casein_138_	NAVPITPTL	Undigested and gastric	*Bubalus bubalis*
Prolyl endopeptidase-inhibitory	P02666	_74_β-casein_81_	VYPFPGPI	Intestinal	*Bos taurus*
Satiety	P02666	_75_β-casein_81_	YPFPGPI	Intestinal	*Bos taurus*

**Figure 4:**
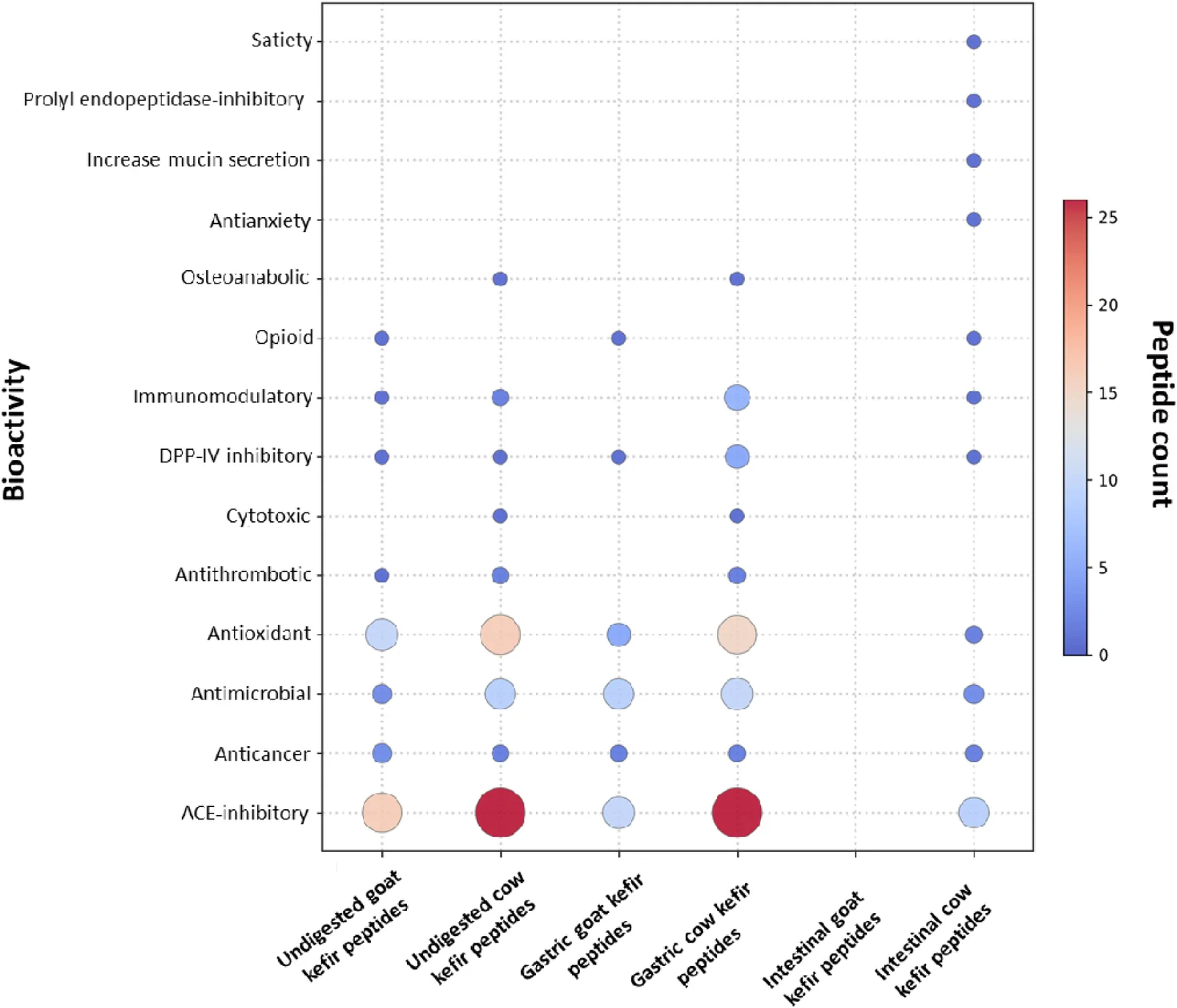
Distribution of bioactive peptides identified in goat and cow kefir before and during *in vitro* gastrointestinal digestion. Bubble plot showing the number of peptides associated with each bioactivity (ACE-inhibitory, anticancer, antimicrobial, antioxidant, antithrombotic, cytotoxic, DPP-IV inhibitory, immunomodulatory, opioid, osteoanabolic, prolyl endopeptidase inhibitory, satiety, increase in mucin secretion, and antianxiety) across undigested, gastric, and intestinal phases. Bubble size and color intensity are proportional to peptide count.

Undigested goat kefir included peptides which have been annotated as angiotensin-converting enzyme (ACE)-inhibitory (16 peptides), anticancer (3), antimicrobial (3), antioxidant (10), antithrombotic (1), dipeptidyl peptidase-IV (DPP-IV) inhibitory (1), immunomodulatory (1), and opioid (1). Undigested cow’s milk kefir included peptides annotated as ACE-inhibitory (24), anticancer (2), antimicrobial (8), antioxidant (16), antithrombotic (2), cytotoxic (1), DPP-IV-inhibitory (2), immunomodulatory (7), and osteoanabolic (1). In the gastric phase, goat kefir had peptides with ACE-inhibitory (10), anticancer (2), antimicrobial (10), antioxidant (5), DPP-IV-inhibitory (1), and opioid (1) activities. The gastric phase for cow kefir had peptides with ACE-inhibitory (24), anticancer (2), antimicrobial (9), antioxidant (15), antithrombotic (2), cytotoxic (1), DPP-IV-inhibitory (5), immunomodulatory (6), and osteoanabolic (1) activities. In the intestinal phase, only peptides from cow kefir were annotated with bioactivity, and these included ACE-inhibitory (9), antianxiety (1), anticancer (2), antimicrobial (3), antioxidant (3), DPP-IV-inhibitory (1), immunomodulatory (1), mucin-stimulating (1), opioid (1), prolyl endopeptidase-inhibitory (1), and satiety-related (1).

The distribution of bioactive peptides, in terms of abundance, varied according to the digestion stage in both goat and cow (Figure 5) milk kefir.

**Figure 5:**
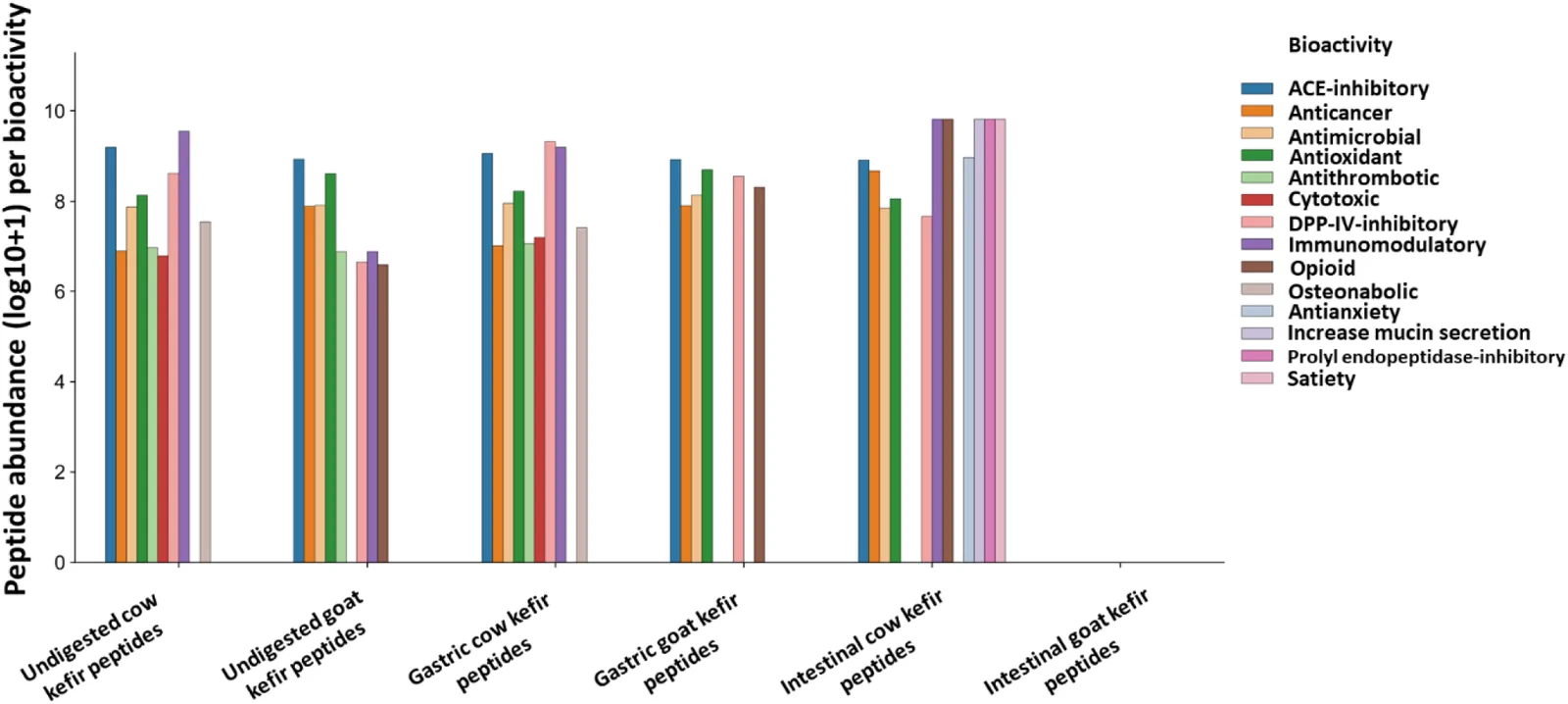
Abundance of bioactive peptides (log 10 transformation) in goat and cow kefir across *in vitro* gastrointestinal digestion. Bar plot illustrating the peptide abundance associated with each bioactivity class in undigested, gastric, and intestinal phases. The data emphasize dynamic changes in peptide release and enrichment during digestion, with distinct patterns observed between goat and cow kefir.

In the undigested samples, peptides exhibiting angiotensin-converting enzyme (ACE) inhibitory activity were highly abundant in both matrices. Additionally, antioxidant and immunomodulatory activities were detected in both kefirs, albeit at lower levels. During the gastric phase, a pronounced increase in the abundance of several bioactive peptide classes was observed, particularly antioxidant, antimicrobial, and immunomodulatory activities. In the intestinal phase, bioactive peptides were identified exclusively in cow kefir, with a substantial increase in the abundance of peptides with immunomodulatory, DPP-IV-inhibitory, and satiety-related activities. The absence of known bioactivity in the intestinal phase for goat kefir likely reflects current limitations in peptide annotation in databases. The sequences identified could have an uncharacterized bioactive potential. This limitation is further compounded by the fact that only 23 unique peptides were identified in the intestinal digestion stage. Such a limited peptide repertoire inherently reduces the likelihood of matching sequences to currently available bioactive peptide databases. Furthermore, intestinal digestion likely promoted extensive peptide hydrolysis, generating shorter peptides and free amino acids that were below the detection and identification limits of the LC-MS/MS workflow employed. Therefore, the absence of annotated bioactive peptides should not be interpreted as evidence of the absence of biological activities. Rather, it reflects both the incomplete annotation of bioactive peptides in current databases and the analytical challenges associated with detecting highly hydrolyzed peptide products generated during intestinal digestion.

Among the different bioactivities identified throughout gastrointestinal digestion, the presence of antioxidant peptides from kefir during digestion could indicate that the consumption of kefir may help prevent or mitigate metabolic processes involved in the onset of diseases and health disorders.^40^ Antimicrobial peptides released from kefir during digestion may help limit the colonization of the gastrointestinal tract by undesirable microorganisms.^41^

### 3.4 Total Antioxidant Capacity (TAC)

#### 3.4.1 Antioxidant Activity of Intact Goat and Cow Kefir before and after Simulated Gastrointestinal Digestion

Undigested goat and cow kefir samples exhibited the lowest antioxidant capacities based on both the ABTS and FRAP assays compared with the gastric and intestinal phases (Table 3). In the ABTS assay, no significant differences were detected between goat and cow kefir (*p* > 0.05), displaying radical scavenging capacities of 40 ± 8% and 35 ± 7%, respectively. Based on the FRAP assay, undigested cow kefir exhibited a higher antioxidant capacity (51.90 ± 6.70 μM TE 100 mL^–1^) than goat kefir (44.04 ± 3.71 μM TE 100 mL^–1^) (*p* < 0.05).

**Table 3:** Total Antioxidant Capacity (TAC) by ABTS and FRAP Assay of Undigested, Gastric, and Intestinal Phases from Goat and Cow Kefir^a^

In vitro digestion phase	Goat kefir	Cow kefir
	ABTS (% scavenging)	
Undigested	40 ± 8^Ab^	35 ± 7^Ab^
Gastric	72 ± 6^Aa^	63 ± 12^Aa^
Intestinal	71 ± 5^Aa^	42 ± 12^Bb^
	FRAP (μM TE 100 mL^–1^)	
Undigested	44.04 ± 3.71^Bb^	51.90 ± 6.70^Ab^
Gastric	53.68 ± 1.67^Ab^	60.48 ± 13.49^Ab^
Intestinal	88.94 ± 34.59^Aa^	88.66 ± 20.99^Aa^

a **TE:** trolox equivalent. Results are expressed as mean ± standard deviation. Different capital letters indicate a significant difference between samples of different species at the same digestion phase. Different lowercase letters indicate a significant difference between the different digestion phases in each sample. A significant difference was considered at a *p*-value <0.05.

During the *in vitro* digestion process, changes in antioxidant capacity were observed. The ABTS radical scavenging activity increased from undigested kefir to the gastric phase for both cow and goat kefir (Table 3). ABTS radical scavenging activity for cow kefir decreased from the gastric phase (63 ± 12%) to the intestinal phase (42 ± 12%), whereas goat kefir retained a high level of antioxidant activity in both the gastric phase (72 ± 6%) and the intestinal phase (71 ± 5%). These findings align partially with those of Carullo et al., who observed that undigested cow kefir exhibited no initial ABTS activity, with significantly increased ABTS activity postdigestion, reaching approximately 300 mg 100 g^–1^ of Vitamin C equivalents.^42^ The enhanced antioxidant capacity observed during gastric digestion may be attributed to dynamic modifications in kefir-derived peptide profiles, suggesting the emergence and contribution of bioactive peptides with yet undiscovered antioxidant properties. The sustained increase in radical scavenging capacity was observed in goat kefir upon digestion. Conversely, the antioxidant capacity by ABTS increased in the gastric phase, followed by a decrease in the intestinal phase for cow kefir, probably attributed to the fragmentation of peptides into amino acids or other peptides that are not capable of performing the antioxidant function.^43^ The observation that goat milk maintains high radical scavenging capacity across digestion may support its potential as a functional food.

Regarding the antioxidant capacity measured via FRAP (ferrous-iron-reducing power), no significant differences were observed between undigested samples and the gastric phase; however, a marked increase was detected in the intestinal phase for both goat and cow kefir. Similar trends were previously reported by Carullo et al., where kefir’s FRAP values increased from approximately 18 mg 100 g^–1^ Fe­(II) equivalents (before digestion) to nearly 350 mg 100 g^–1^ (after intestinal static *in vitro* digestion).^42^ Yilmaz-Ersan et al. observed a significant increase from the undigested kefir (0.57 ± 0.075 mg TE 100 mL^–1^; ∼22.8 ± 3.0 μM TE 100 mL^–1^) to the gastric phase (2.49 ± 0.184 mg TE 100 mL^–1^; ∼99.5 ± 7.4 μM TE 100 mL^–1^), followed by a decrease in the intestinal phase (albeit still higher than undigested kefir, 1.34 ± 0.010 mg TE 100 mL^–1^; ∼53.5 ± 0.4 μM TE 100 mL^–1^).^44^ The increased reducing power may also result from the release of bioactive peptides, produced by LAB or generated during digestion.^45,46^

#### 3.4.2 Antioxidant Activity of Goat and Cow Kefir Peptide Extracts before and during Simulated Gastrointestinal Digestion

Peptides extracted from undigested goat and cow kefir samples showed antioxidant activity in all assays (Table 4). In the ABTS assay, no statistical difference (*p* > 0.05) was observed between the undigested kefir of different species. However, undigested cow kefir had a higher antioxidant capacity than undigested goat kefir for the FRAP assay (19.86 ± 0.96 μM TE 100 mL^–1^ vs 16.72 ± 1.36 μM TE 100 mL^–1^) and the DPPH assay (96% vs 89% radical scavenging activity).

**Table 4:** Total Antioxidant Capacity (TAC) by ABTS, FRAP, and DPPH Assay of Undigested, Gastric, and Intestinal Phases from Goat and Cow Kefir Peptides[Table-fn tbl4fn1]

*In vitro* digestion phase	Goat kefir peptides	Cow kefir peptides
	ABTS (% scavenging)	
Undigested	39 ± 3^Ab^	40 ± 3^Ab^
Gastric	89 ± 7^Aa^	73 ± 2^Ba^
Intestinal	91 ± 2^Aa^	81 ± 8^Ba^
	FRAP (μM TE 100 mL^–1^)	
Undigested	16.72 ± 1.36^Ba^	19.86 ± 0.96^Ab^
Gastric	4.76 ± 1.72^Ac^	6.42 ± 1.46^Ac^
Intestinal	10.92 ± 3.50^Bb^	31.28 ± 1.63^Aa^
	DPPH (% scavenging)	
Undigested	89 ± 1^Ba^	96 ± 2^Aa^
Gastric	52 ± 5^Bc^	64 ± 5^Ab^
Intestinal	61 ± 3^Ab^	47 ± 6^Bc^

i **TE:** trolox equivalent. Results are expressed as mean ± standard deviation. Different capital letters indicate a significant difference between samples of different species at the same digestion phase. Different lowercase letters indicate a significant difference between the different digestion phases in each sample. A significant difference was considered at a *p*-value <0.05.

In the ABTS assay, the radical scavenging activity of peptide fractions from both undigested kefir types was lower than the peptide fractions after *in vitro* digestion (Table 4). In both the gastric and intestinal phases, goat kefir peptides exhibited higher antioxidant capacity than cow milk kefir peptides (*p* < 0.05, Table 4). In the FRAP assay, antioxidant activity decreased significantly after gastric digestion in both kefir types and increased after intestinal digestion. No differences in FRAP values were observed between goat and cow milk kefir peptides during the gastric phase. After intestinal digestion, cow kefir peptides had higher FRAP values than those from goat kefir (*p* < 0.05), which may reflect differences in peptide composition.

Regarding DPPH radical scavenging activity, undigested peptide extracts from goat and cow kefir showed high initial activity (89 ± 1% and 96 ± 2%, respectively), and significantly lower levels after gastric digestion (52 ± 5% and 64 ± 5%, respectively) and intestinal digestion (61 ± 3% and 47 ± 6%, respectively). For goat milk kefir, the DPPH radical scavenging activity was lower in the gastric phase than in the intestinal phase. Contrary with our findings, Shu et al. reported that peptides extracted from fermented goat milk (not kefir) subjected to *in vitro* gastrointestinal digestion exhibited a higher antioxidant activity profile by DPPH assay in the gastric phase compared with the intestinal phase.^47^

The data indicate that gastrointestinal digestion modulates the antioxidant potential of kefir-derived peptides. Hydrophobic and aromatic amino acids (e.g., proline, histidine, tyrosine, and tryptophan) likely contribute to peptide antioxidant activity via direct hydrogen or electron transfer.^48–50^ Additionally, hydrophobic residues at the C- and N-terminal regions have been associated with greater antioxidant capacity.^51^ These sequence-related characteristics were observed in peptides identified from both goat and cow milk kefir. Gastric digestion markedly increased the abundance of peptides enriched in aromatic and hydrophobic residues, particularly YQKFPQY and VYPFPGPIPN, whose tyrosine-, phenylalanine-, and proline-rich sequences have been associated with antioxidant activity. YQKFPQY increased from 1.04 × 10^7^ to 5.18 × 10^9^ in goat kefir and was detected exclusively during the gastric phase in cow kefir (1.34 × 10^9^). Likewise, VYPFPGPIPN increased from 3.06 × 10^7^ before digestion to 1.24 × 10^9^ after gastric digestion in cow kefir, before declining to 9.72 × 10^7^ following intestinal digestion.

### 3.5 Peptides Exerting Antimicrobial Effects

The antimicrobial effect of peptides extracted from goat and cow kefir was evaluated via growth inhibition assays for two bacteria, Gram-negative and Gram-positive, *E. coli* and *S. aureus*, respectively (Figure 6A and 6B).

**Figure 6:**
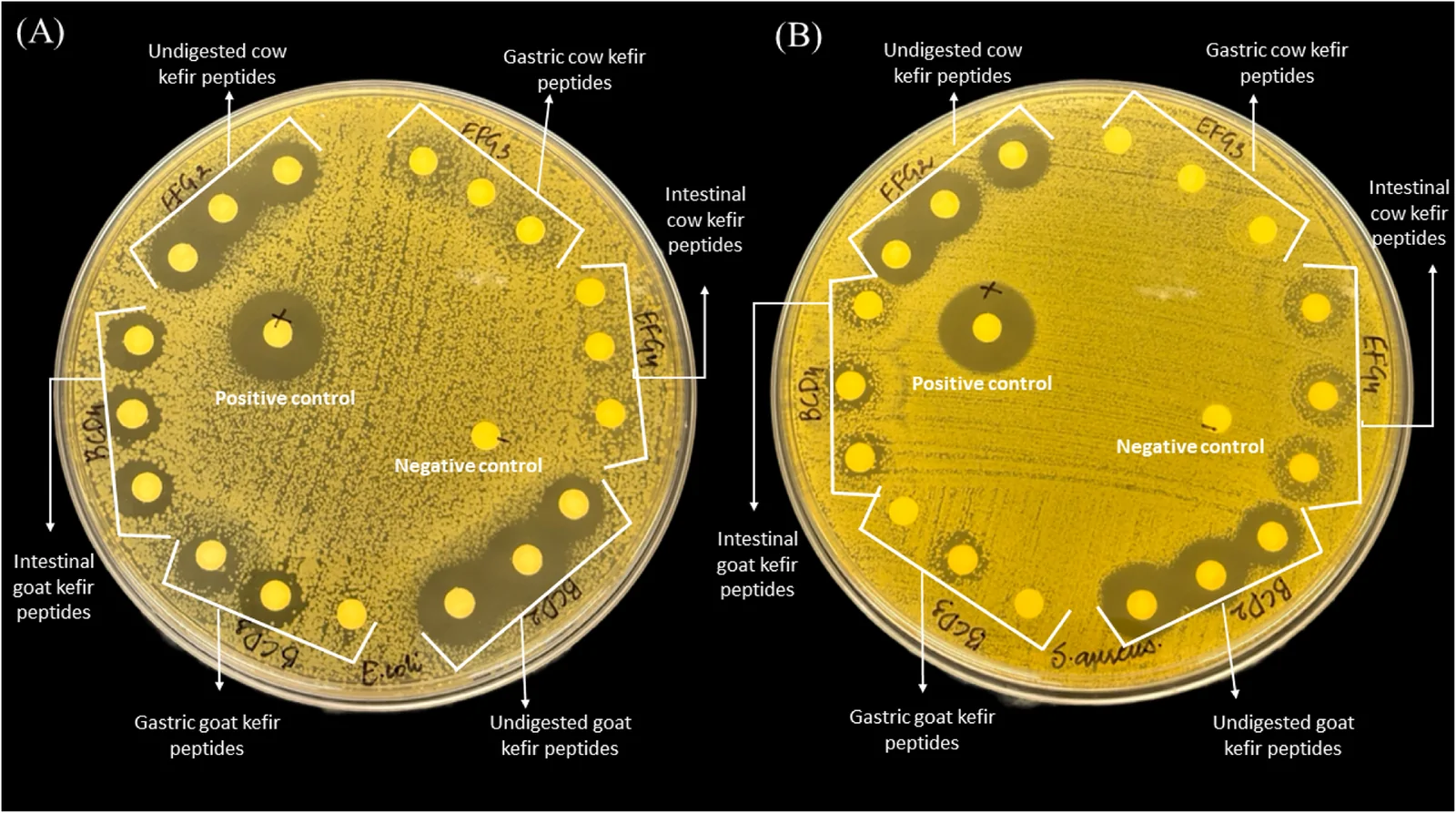
Antimicrobial activity of peptides extracted from goat and cow kefir across undigested, gastric, and intestinal phases against *E. coli* (A) and *S. aureus* (B). Streptomycin was the positive control, and PBS was the negative control. BCD2: undigested goat kefir peptides; BCD3: gastric goat kefir peptides; BCD4: intestinal goat kefir peptides; EFG2: undigested cow kefir peptides; EFG3: gastric cow kefir peptides; EFG4: intestinal cow kefir peptides.

The antimicrobial activity of peptides from the different samples against the two tested bacterial strains differed according to the digestive stage. Although the same sample volume (40 μL) was used in the antimicrobial assay, the total amount of peptide, determined by the BCA assay, varied among the digestion stages. Intestinal digestion increased the peptide content of goat milk kefir but decreased that of cow milk kefir relative to the undigested samples. While the undigested kefirs exhibited similar inhibitory effects against *E. coli* (with inhibition zones of 17 mm and 15 mm, respectively) (Table 5), the higher peptide content in intestinally digested goat milk kefir may have contributed to its enhanced antimicrobial activity. Conversely, the lower peptide content in intestinally digested cow milk kefir may have partially accounted for the absence of antimicrobial activity against *E. coli*, although differences in peptide composition and sequence are also likely to influence the antimicrobial response. In addition, peptides from goat and cow kefir before digestion inhibited the growth of *S. aureus*; however, the gastric phase did not inhibit *S. aureus* growth at the levels tested herein. Notably, although the peptides generated during the intestinal phase were unable to completely inhibit the growth of *S. aureus*, a residual antimicrobial effect was still observed, as evidenced by the presence of bacterial colonies within the inhibition zones of both goat and cow milk kefir intestinal digests.

**Table 5:** Antimicrobial Activity of Peptides Extracted from Goat and Cow Kefir during Undigested, Gastric, and Intestinal *In Vitro* Digestion Steps against *Escherichia coli* and *Staphylococcus Aureus* [Table-fn tbl5fn1]

			Inhibition zone (mm)	
Sample/Control	Total amount peptide (mg)[Table-fn tbl5fn2]	*In vitro* digestion stepIn vitro digestion step	*E. coli* ATCC 25922	*S. aureus* ATCC 29213
Goat kefir peptide	8	Undigested	17^a^	16^b^
	4	Gastric	7^bc^	0^e^
	25	Intestinal	14^ab^	10^d^ (weak zone inhibition with bacterial colonies within zone)
Cow kefir peptide	15	Undigested	15^ab^	16^b^
	2	Gastric	0^c^	0^e^
	2	Intestinal	0^c^	13^c^ (weak zone inhibition with bacterial colonies within zone)
	Antibiotic concentration (mg mL^**–1**^)			
Streptomycin	50	[Table-fn tbl5fn3]	21^a^	20^a^
Negative control (PBS)	[Table-fn tbl5fn3]	[Table-fn tbl5fn3]	0	0

i Results are expressed as the mean of the zone of inhibition (mm). Different letters within each column indicate a significant difference between means at a significance level of 0.05.

ii 40 μL of the samples was transferred onto the paper disk. The total amount of peptide was measured using the BCA assay.

iii Absence of concentration values or an *in vitro* digestion step.

Though studies evaluating the effects of peptides extracted from goat and cow kefir before and after *in vitro* digestion are scarce in the literature, some studies have investigated the antimicrobial activity of peptides from several dairy products, including kefir and yogurt. Sodanlo and Azizkhani (2021) found that water-soluble peptides (20 mg/mL) extracted from cow kefir inhibited the growth of *E. coli* and *S. aureus* over a 28-day storage period.^52^ Ashokbhai et al. found that sheep milk fermented with *Lactobacillus fermentum* KGL4 inhibited the growth of several microorganisms, including *E. coli* MTCC 1687, during different time periods (12 to 48 h), and the inhibition zone ranged from 12.33 ± 0.58 to 13.00 ± 0.00 mm.^53^

Hydrolysis during digestion leads to the release of new peptides with bioactive properties distinct from those found in the undigested samples,^54^ which may decrease antimicrobial activity against bacteria. Furthermore, the differences in antimicrobial activity observed between *E. coli* and *S. aureus* may be partially explained by the distinct cell envelope structures of Gram-negative and Gram-positive bacteria. Gram-negative bacteria possess an outer membrane rich in lipopolysaccharides, whereas Gram-positive bacteria have a thicker peptidoglycan layer but lack an outer membrane. These structural differences influence peptide–membrane interactions and, consequently, bacterial susceptibility to antimicrobial peptides, which may account for the different inhibitory responses observed after gastrointestinal digestion. Although the exact mechanism for antimicrobial peptides may be unclear, they generally have two physical attributes in common: a cationic charge and a high proportion of hydrophobic residues.^55^ These peptides typically form cationic amphipathic α-helices or β-sheets that interact electrostatically with anionic bacterial membranes, leading to membrane disruption through mechanisms such as the carpet, toroidal pore, or barrel-stave models.^56,57^ Additionally, peptides may enter microbial cells via lipid-assisted or receptor-mediated translocation,^58^ where they interfere with essential cellular processes, including DNA and protein synthesis, protein folding, enzymatic activity, and cell wall formation.^57,59^

This study reveals that kefir microorganisms partially hydrolyze milk proteins, leading to the release of numerous bioactive peptides in the undigested samples. Furthermore, *in vitro* digestion of goat and cow milk kefir results in the release of different bioactive peptides. Goat milk kefir can provide a similar array of bioactivities as cow milk kefir (e.g., antioxidant and antimicrobial activities, ACE- and dipeptidyl peptidase IV (DPP-IV) inhibitory potential). Moreover, new peptides with bioactivity have been identified in kefir during the intestinal phase (e.g., antianxiety, satiety, and mucin production). The kefirs and their extracted peptides exhibited antioxidant activity across digestion, which may indicate they could benefit the consumer. The presence of antimicrobial activity across digestion from goat milk kefir could provide a consumer benefit by helping to prevent pathogen growth. Whether peptides produced from kefir during digestion are absorbed systemically remains unclear. If peptides are absorbed, additional functions of peptides may be relevant to consumer health, such as antihypertensive actions for the management of hypertension.

## Supplementary Material





## Notes

; I The Supporting Information is available free of charge at https://pubs.acs.org/doi/10.1021/acs.jafc.6c08235.

Simulated gastric and intestinal fluid used for *in vitro* digestion­( Supplementary Table 1)­(PDF)
Identification of protein and peptides across *in vitro* digestion by LC-MS/MS (XLSX)
