# Supplementary material for: Peptidomic Characterization and Functional Properties of Goat and Cow Milk Kefir before and after In Vitro Digestion
Source: J Agric Food Chem. 2026 Aug 11;74(33):26625–39. doi: 10.1021/acs.jafc.6c08235 (PMC13523750; doi:10.1021/acs.jafc.6c08235)
Supplement: Supplementary file 1 [file jf6c08235_si_001.pdf]

Supporting Information

Peptidomic characterization and functional properties of goat and cow milk kefir before and after *in vitro* digestion

Rayssa Cruz Lima [a, b], Bum Jin Kim [c], Carini Aparecida Lelis* [b], Brianne Wai [c], Rohit Kumar [c], Jelmir Craveiro Andrade [b], Marie Biondi Ryan [c], David C. Dallas [c], Carlos Adam Conte-Junior* [a, b]

[a] *Graduate Program in Veterinary Hygiene (PPGHV), Faculty of Veterinary Medicine, Fluminense Federal University (UFF), Vital Brazil Filho, Niterói, RJ, 24230-340, Brazil*

[b] *Center for Food Analysis (NAL), Technological Development Support Laboratory (LADETEC), Federal University of Rio de Janeiro (UFRJ), Cidade Universitária, Rio de Janeiro, RJ, 21941-598, Brazil*

[c] *Nutrition Program, School of Nutrition and Public Health, College of Health, Oregon State University, Corvallis, OR, United States*

* Corresponding author. Center for Food Analysis, Federal University of Rio de Janeiro (UFRJ), Cidade Universitária, Rio de Janeiro, RJ, 21941-901, Brazil. E-mail address: carinilelis@iq.ufrj.br; carinilelis@gmail.com (Carini Aparecida Lelis); conte@iq.ufrj.br (Carlos Adam Conte-Junior)

Supplementary Table 1. Simulated gastric and intestinal fluid used for *in vitro* digestion.

Item	Stock concentration (M)	Amount needed (mL)
Simulated gastric fluid (SGF, pH 3, 400 mL final volume)		
KCl	0.5 M	6.9 mL
KH_2PO_4	0.5 M	0.9 mL
$NaHCO_3$	1 M	12.5 mL
NaCl	2 M	11.8 mL
$MgCl_2(H_2O)_6$	0.15 M	0.4 mL
$(NH_4)_2CO_3$	0.5 M	0.5 mL
HCl	6 M	1.3 mL
Milli Q water	-	365.7 mL
Simulated intestinal fluid (SIF, pH 7, 400 mL final volume)		
KCl	0.5 M	6.8 mL
KH_2PO_4	0.5 M	0.8 mL
$NaHCO_3$	1 M	42.5 mL
NaCl	2 M	9.6 mL
$MgCl_2(H_2O)_6$	0.15 M	1.1 mL
HCl	6 M	0.7 mL
Milli Q water	-	338.5 mL
